# Countermeasures to Avoid Noncooperation in Fully Self-Organized VANETs

**DOI:** 10.1155/2014/589563

**Published:** 2014-06-26

**Authors:** Jezabel Molina-Gil, Pino Caballero-Gil, Cándido Caballero-Gil

**Affiliations:** Faculty of Mathematics, University of La Laguna, 38271 Tenerife, Spain

## Abstract

The secure and efficient exchange of information in vehicular ad hoc networks (VANETs) involves more challenges than in any other type of ad hoc networks. This paper proposes a new vehicular communication system based on mobile phones for fully distributed and decentralized networks. In these networks, communications depend on individual nodes, which could decrease the efficiency and reliability of transmitted information. Besides, the limitation in the resources of mobile devices is an additional obstacle in the forwarding problem, and the content of the information generated by individual nodes must be considered inherently unreliable. In particular, this paper proposes the application of groups as a basis structure for a cooperation mechanism useful in event generation and in packet retransmission. Its aim is to promote the involvement of nodes in network performance. Given that such participation involves consumption of node resources, a group-based structure is here used not only to reduce communication overload but also to prevent sending false information and to encourage nodes in relaying packets. Several simulations of the proposal have been done, and the results have confirmed that this is a promising approach to increase network efficiency and trust in transmitted information, while reducing the number of selfish nodes in VANETs.

## 1. Introduction

Vehicular ad hoc networks (VANETs) have been proposed as a solution based on intervehicles communication to prevent adverse circumstances on the roads and to increase efficiency in traffic management. In order to turn this type of networks into a reality that helps to improve road safety, several security communication tools are necessary to protect them from many possible types of attacks such as attacks to jeopardize the connectivity and attacks to modify forwarded information.

The main goal of this paper is the proposal of new tools that allow the protection of VANETs against such attacks, ensuring as far as possible that generation and retransmission of information are done correctly. This paper, based on two previous works of the authors [1, 2], includes an analysis of various cooperation mechanisms needed to deploy a reliable and functional VANET where the nodes are fully autonomous and independent. It describes different techniques for promoting cooperation. Specifically, different strategies to motivate nodes to participate in relaying packets properly and to ensure greater availability and quality of the network are here presented. Besides, it deals with the need to protect the content of the relayed information by using reactive groups that ensure the accuracy of the information without involving any significant delay. In order to reduce the time required to generate the information, a group-based structure is proposed to handle more efficiently the generated packets.

The rest of the paper is organized as follows. Related work is overviewed in Section 2.  Section 3 describes secure event generation through cooperative groups and includes an analysis of implementation results from simulations.  Section 4 addresses relevant cooperation factors in packet forwarding. Finally, conclusions are given in Section 5.

## 2. Related Work

Research on cooperation in VANETs can be classified mainly into two categories according to the way of encouraging participation of nodes in the network: through incentives or through reputation. In incentive-based systems, nodes pay and charge for participating in the network. The work in [3] proposes the use of virtual credit in incentive schemes to stimulate packet forwarding in general mobile ad hoc networks. A system where vehicles receive an incentive for forwarding and carrying advertisements is described in [4]. Besides, [5] discusses some unique characteristics of incentive schemes specifically thought for VANETs and focuses on a receipt-based reward scheme. On the other hand, in reputation systems, malicious nodes are detected and isolated from the network. In [6], an event-based system is used to prevent nodes from spreading false traffic messages by determining whether incoming traffic messages are significant and trustworthy to the driver. The work in [7] describes a mechanism for detecting possible malicious nodes through the use of three different modules whose sum determines node reputation. The proposal called VARS [8] uses direct and indirect trust as well as appended opinions to enable confident decisions on event packets. Another interesting reputation system is described in [9], where trust relationships and packet-acceptance decisions are based on instant observation and relaying behaviour of nodes.

However, all the aforementioned tools, including the reputation system proposed in [10], require certification authorities, which are responsible for delivering public/private keys and certificates. In particular, [11] proposes that a regional transportation authority plays such a role. Therefore, none of those solutions can be considered applicable to fully distributed and decentralized networks such as the ones discussed in this work. The closest references to this paper are [12, 13], where cooperative groups coordinate actions taken by multiple vehicles to make optimal decisions. Reference [14] proposes a novel approach to compose a trustworthy group in a P2P model, which is fully distributed and scalable. On the other hand, the work in [15] uses routing for communications and introduces cooperation as a service, based on a cluster structure. Finally, [16] proposes a flocking scheme for a group of vehicles, which focuses on their decentralized coordination so that they can cooperate in complex environments.

Many references on VANETs discuss the problem of packet forwarding. Those works assume the existence of a sender and a receiver because the routing issue where intermediate nodes are used to perform connections between any pair of nodes is one of the most basic networking issues in ad hoc networks. Reference [17] classifies routing protocols into single-black hole attack and collaborative-black hole attack, analyses the categories of these solutions, and provides a comparison table. A remarkable result of the research is a tool for driving assistance that allows creating a real and safe vehicular ad hoc network by using only smartphones called VAiPho (VANET in Phones) [1, 2]. This scheme offers the possibility of deploying a real and secure VANET quickly and economically. In VAiPho, no specific routing protocol is required because broadcast is used to disseminate information.

## 3. Cooperative Groups

Most proposals to improve security in VANETs use cryptographic tools such as authentication, key management, or pseudonyms. However, these schemes do not provide any solution to ensure that event information generated within the network is true, which means that, even if all nodes are cooperative and forward messages, it is possible that such messages are announcements of false events. In this section, we analyse the possibility of using cooperative groups to prevent generating and forwarding false announcements. A group is here defined as a set of vehicles that are located in a close geographical area. Many papers propose the formation of groups to avoid overloading the network by reducing communication overload, and so forth. In [18], the authors propose to divide roads into cells so that groups are formed by vehicles within the same cell. However, the tasks of dividing a highway into cells by using GPS to determine the group of each vehicle and choosing a leader for each group are time-consuming tasks and require heavy computation. Thus, they are nonappropriate in fully self-organized VANETs.

The proposal here presented uses the idea of reactive group formation to generate events cooperatively so that groups are formed when an event is detected. This not only allows improving the reliability of transmitting information, but also reduces channel overload because the number of generated packets decreases.

### 3.1. Security Mechanisms

Since our proposal is self-organized, the device associated to each network node *n* must be able to generate a pair of private and public keys in a decentralized way. In order to obtain a signed certificate for its public key, it contacts with several legitimate network nodes and if these nodes confirm that node *n* is trustworthy, a certificate with its private key is generated. Like what was presented in [19], a similarity can be found between the proposed security mechanisms and social networking sites like Facebook, Twitter, and so forth.

The system asks the new node for information about its contacts in some social network and looks for registered and trustworthy nodes in the system. The results are showed to the new node, which selects the nodes to sign its certificate. During the signing process, the system exchanges the generated certificates so that each user holds the signature generated by itself and the signatures produced by its friends.

### 3.2. Group Formation and Leader Election

An important issue about the formation of groups is the choice of the group leader and the nodes that belong to each group. The lack of centralized structures and the dynamism of the environment imply a complex challenge.

**Figure 1:**
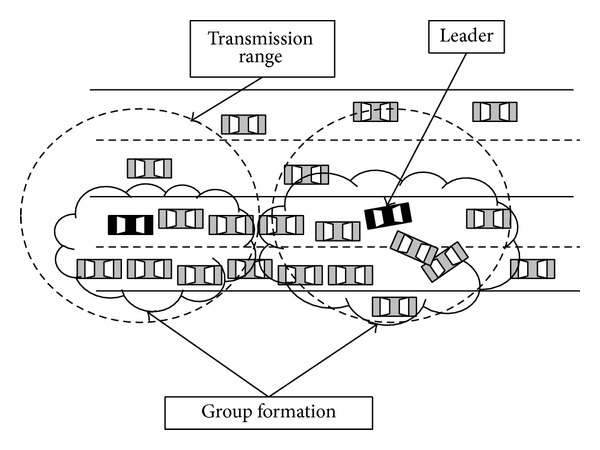
Group formation and leader election.

When a node detects an incident, it sends a message to all nodes within its transmission range, to form a reactive group. This node will be the leader of the group (see Figure 1). *G* denotes a group of nodes *n*_*i*_ with location loc⁡(*n*_*i*_, *T*), which are within the leader's transmission range *R* at the time *T* when it detects an incident:

(1) $$\begin{array}{c} N=\left\{n_{1},n_{2},n_{3},\ldots \right\},\\ G=\left\{n_{i}^{}\in N;\mathrm{loc}\left(n_{i}^{},T\right)\leq R\right\}. \end{array}$$

During group formation, three different types of messages can be distinguished.

- *Detection Message*. It is sent by a node when it detects an incident, in order to form a group. It contains information used by nodes to determine whether the event is true. It will be detailed in the next subsection. Nodes receiving it will assume that the origin node is the group leader.
- *Signature Message*. It is signed by neighbouring nodes and sent to the group leader in response to a detection message, indicating agreement with received data.
- *Event Message*. It is sent by the group leader to all nodes, containing all the received signatures and the incident information. This message is stored and broadcast by all receiving nodes.

It is possible that two or more nodes begin the group formation process. This situation could produce more than one leader in a group. The solution to this problem can be reached through a cooperative decision between leaders. In particular, each node that generates a detection message must store the timestamp of the generation. If any node within its transmission range receives this packet and has previously generated an identical packet, meaning that it is another potential leader, it compares both packet timestamps. In this way, the oldest one corresponds to the leader. This solution has been tested in VAiPho implementation with successful results.

### 3.3. Event Generation

Before sending information about an event that has been detected, a verification process is necessary to prevent forwarding fraudulent information. This process is performed in three steps, which correspond to the three types of messages described above. This process is part of the proposed scheme and is performed within each group as described below.

The proposal allows nodes inside a group to decide whether some received data are true or not, by matching them with the perceived information. If both pieces of information match, the node signs the received message indicating that it agrees with the content of the message. Thus, in order to generate an event, all vehicles that are part of the cooperative group must agree with the information they receive from their neighbourhood. The protocols to make this process would imply an overload of the channel and a significant delay of communications if no mechanism to minimize them was adopted. In our proposal, each vehicle processes locally the received events before making a decision based on a three-stage sequential scheme. The first one is the decision-making stage, corresponding to detection and signature message, where each vehicle locally processes the message received about an event from the leader, verifies it, and compares it with the information it receives directly from its environment. The second stage is data aggregation. Once the leader concludes that it has received enough signed messages about the existence of an event, it applies an aggregation method to combine them. This process was described in [20]. Finally, an event message is generated and forwarded through the network to reach as many users as possible.

In order to decide whether the received data match the information perceived by the receiver, the proposed decision-making stage is based on four dimensions: time, location, direction of travel, and speed. Firstly, the time interval in which an event is valid must be taken into account. Secondly, the location of an event, on the road or on the map, is an essential parameter. Finally, the direction of travel in which the event is located must be distinguished due to the usual existence of two-way traffic. Additionally, since VAiPho can detect traffic jams, another interesting parameter is speed. Thus, in the decisions-making stage, each node follows rules based on the above four parameters.

The first verification is the date of the event. If it is an expired event, the node drops the packet. The second test parameter is the location of the event. The node checks the coordinates (*X*, *Y*, *Z*) of the received packet and compares them with its current coordinates (*X*, *Y*, *Z*). If the vehicle is within the event range, it must be able to detect the event. The third test is the direction of travel. In two-way traffic roads, a traffic jam is possible in a direction where the opposite direction is completely free of vehicles. In order to do this test, nodes must verify the direction of travel included in the packet and compare it with their direction of travel. Finally, a speed check is performed. If a node receives a packet reporting the existence of a traffic jam on the road in which it is circulating, the node checks for a period of time if its speed (*Speed*_*current*_) is below the maximum speed of the road (*Speed*_max⁡_) divided by a factor, which in the implementation was equal to 4. If so, it concludes that there is a traffic jam:

(2) if  Speedcurrent≥Speedmax⁡4, there  is no  traffic  jam,otherwise, there is  a traffic  jam.

In the last step, after receiving enough of signed messages, the leader adds all the signatures, generates an event message, and sends it to all nodes within its transmission range. At this moment, all nodes that receive this message must validate the information through the verification of signatures. Nodes that receive this validated event store it in their database and follow the store-and-carry paradigm. In this way, they forward the packet within the network and prevent the spread of false information.

### 3.4. Performance Evaluation

In order to analyse how fast and effective the event generation module is and how the proposed group-based structure affects the implementation, several NS-2 simulations have been done based on data got from a real device implementation. This section presents some details and results obtained by averaging 100 simulations using different network sizes over the same area of 2000 square meters, that is to say, considering different situations regarding traffic density. Simulations were done in networks between 1000 and 10000 nodes. The most relevant parameters selected for the simulation have been given as follows: total number of lanes for each direction = 3, simulation time = 1000 s, moment when motions start = 0 s, moment when relaying begins = 40 s, transmission period = 10 s, transmission range = 100 m, and travelled distance before the event happens = 800 m. Speeds and directions of nodes in the NS-2 simulations were random.

The main goal of the simulations was to evaluate, on the one hand, the number of generated packets using reactive groups, and, on the other hand, the effects of our proposal in the computational complexity, that is to say, the time our cooperative mechanism takes to warn all network nodes about the existence of an event. For the purpose of these simulations, we used and compared the proposal with a basic scheme without any cooperation mechanism, where each node that detects or receives an event sends it to all nodes in its transmission range.

**Figure 2:**
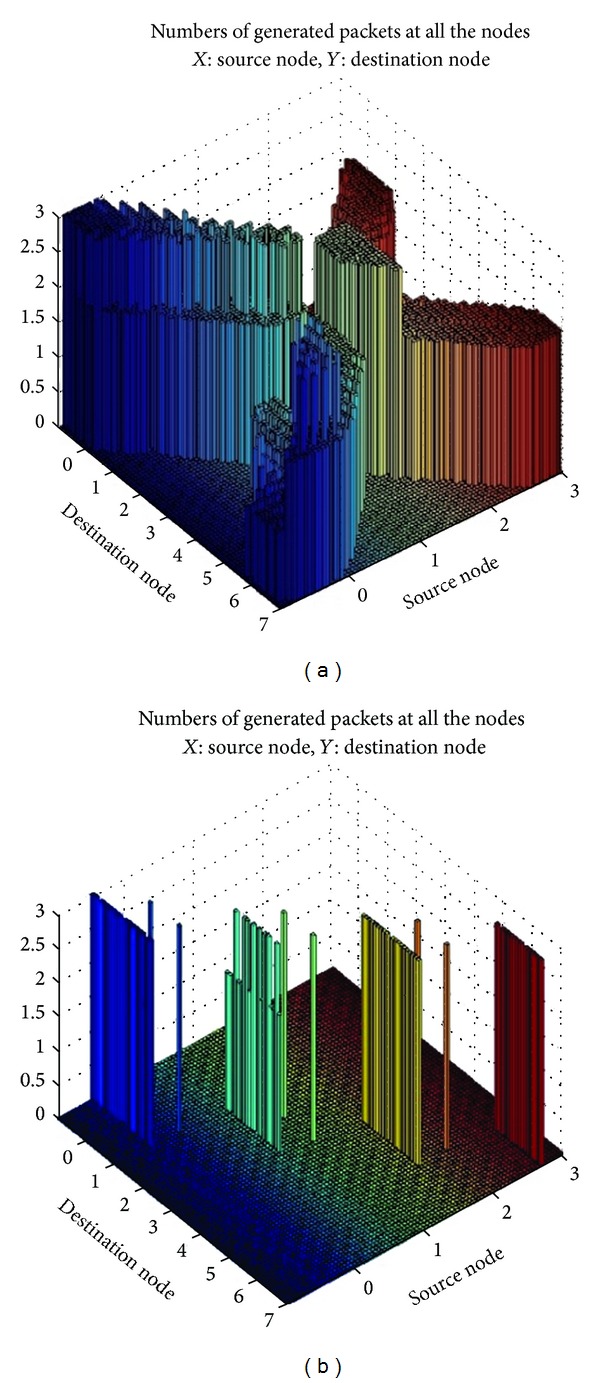
Number of generated packets with and without cooperation groups.

The first simulations correspond to a traffic jam and the corresponding warning packet forwarding with and without reactive groups.  Figure 2 shows that the number of generated packets in the simulation without groups is much higher than when using reactive groups, even though in this case such a number includes the packets generated by the group formation algorithm. The decrease in the number of generated packets allows making better use of the channel. Thus, Figure 2 shows how using reactive groups can reduce the number of generated packets and improve the channel usage.

**Figure 3:**
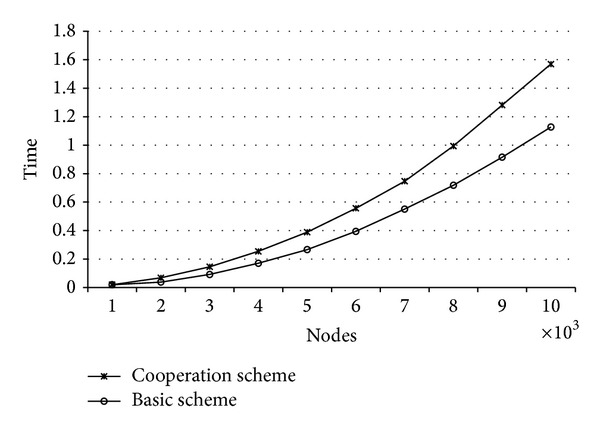
Time cost.

Connections between vehicles in VANETs are usually short, so any proposed mechanism requiring communication between vehicles has to be fast enough to prevent data loss in communications. In Figure 3, we can see the impact of node density in time cost of communication both with the basic scheme and with the proposed cooperation scheme. We can conclude that the use of the group-based mechanism does not involve a significant increase in time cost of management when the packet is received.

The difference between the basic scheme and the cooperation scheme depends on the computational cost required to form the group structure when a new event is detected. However, the reduction in the number of generated packets involves a relevant communication improvement. Another sensitive aspect of the implementation is that nodes must check the accuracy of the received information in order to avoid attacks. This leads to the verification of some signatures, which explains the delay it causes. However, this delay does not involve a significant increase in time. Finally, when the number of nodes increases, the time for processing packets also increases.

## 4. Cooperation in Packet Forwarding

### 4.1. The Problem

In order to spread information, VAiPho users relay packets to all the vehicles in the same group. This implies an important resource consumption, which in the specific case of VAiPho involves the problem of using devices with limited battery. Furthermore, in VANETs, nodes can move at very high speeds, so links can be intermittent. This requires that, according to the store-and-carry approach [5], nodes store the events and exchange them when they meet other vehicles, which could produce also a problem of storage space.

**Figure 4:**
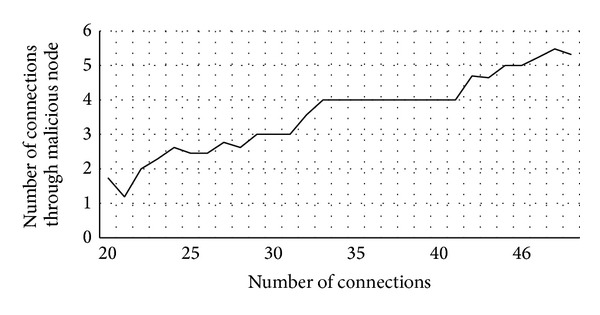
Connections with 5% of malicious nodes.

As we see below, if the number of malicious users who do not cooperate in forwarding packets is not high compared to the total number of users, they will not pose a big threat to the network.  Figure 4 shows the effect of these users in the performance of the network. In particular, we have simulated a network with 100 nodes where around 5% of them do not forward packets. Nodes in the simulations move randomly, and communications between them are also random, making around 20 and 50 connections for each simulation. From the obtained data, we estimate that the probability of meeting a malicious node is 12% in the worst case. Therefore, under these conditions, malicious nodes cannot be considered a major threat to the proper performance of the network. However, VAiPho users might choose not to relay packets or to turn off their devices to save power, and this would seriously affect the network because these vehicles would no longer be part of the network. Indeed, the greater the number of nodes that collaborate in forwarding packets, the better the performance of the VANET.

Given the aforementioned limitation of battery and storage of mobile phones, it is possible that nodes try to act passively by only receiving information from the network rather than storing and forwarding packets. These users would benefit from information relayed in the network without participating in it, so they passively damage and degrade network performance, endangering its connectivity.

Hence, a specific module is necessary in VAiPho to encourage nodes in network cooperation. Users must be motivated to keep their devices turned on and forward packets properly, which is accomplished by offering a new incentive scheme to encourage participation of nodes as part of VAiPho. This aim deals with two factors that may influence packet forwarding due to selfish and/or passive behaviour. One of these factors is battery consumption, which can affect packet forwarding during sending packets to inform neighbouring nodes. Another factor is the limitation of the storage space, which is required to store all the events that are generated and/or received.

### 4.2. Forwarding Cooperation

The proposed protocol uses the idea of reactive groups where nodes in the same transmission range join together in the same group. This idea of groups allows reducing the number of packets in the network because the leader is in charge of making decisions about the packets that reach its group. This prevents from having multiple retransmissions of the same packet.

When a node detects a traffic jam, it sends the information, and the nearby receiving vehicles start to disseminate it to other vehicles while they are moving by using the leaders of the groups. These packets are forwarded for a certain period of time and distance from the source provider.

**Figure 5:**
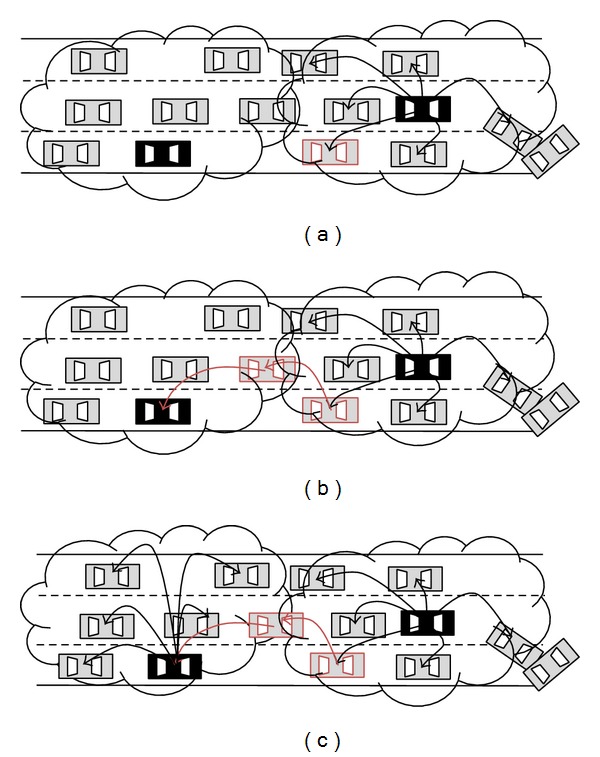
Packet forwarding.

For this type of packets, the detector node sends the packet to all vehicles that are in its scope. It will be the leader of the group in its range of coverage; see Figure 5(a). In this case, the number of generated packets is relatively high because the aim of such packets is to provide information to as many vehicles as possible. If all vehicles were devoted to covering this type of packets without any control, the network would be overloaded. Although the number of generated packets is large, thanks to the idea of groups, the leader manages to relay them in an orderly manner. The leader will receive the packet and will be responsible for broadcasting it within its group. The result is that no nodes will receive the same packet many times from its neighbours because if a leader receives a packet that had been previously received, it will not relay it again to its group. Moreover, the leader will seek a route within its group to a neighbour group through one of the members of its group nodes (see Figure 5(b)). Hence, it achieves a reduction of both the number of retransmissions between groups and the number of retransmissions inside the group (see Figure 5).

In this proposal, both the leader of the group and the node selected to send the packet to other groups consume more resources than other vehicles inside the group. For this reason, an incentive scheme is necessary to encourage the nodes to be leaders of the groups and to relay information to other groups when they are chosen to do it. In order to reach this aim, a new incentive scheme is proposed inspired by a micropayment scheme [21], where each payment for a forwarding service can be thought as a lottery ticket where, for example, the award could be gas vouchers.

As we are going to explain below, nodes relaying packets will receive a lottery ticket. This ticket allows that both the authority and the winner node can determine whether it is a winning ticket or not. Besides, this method encourages relaying packets because the prizes will not be only for the node with the winning ticket but also for the node that received the forwarded packet.

#### 4.2.1. Incentive Scheme

The presented model proposes a scheme based on a type of lottery in which each node has a non-null probability of winning. We denote by *n* the current node, *N*_*i*_ the child nodes that *n* broadcasts the packet. The node that detects an event generates a packet that contains a unique identifier* PackID,* the information* Information,* and a hash code *H* computed randomly with a certain size:

(3) $$\begin{array}{c} \left[PackID\;|\;Information\;|\;H\right]. \end{array}$$

When a node *n* receives the packet, it checks the information. If *n* decides to participate in the forwarding, it sends the message to other nodes and waits for a receipt *rec*_*Ni*_ justifying that it sent the packet to their children nodes *N*_*i*_. Then, the node *n* computes for each child node a receipt *rec*_*Ni*_ with a hash on *PackID*, *NodeID*_*N*_ y *rec*_*Ni*_ and checks the result against *H*:

(4) $$\begin{array}{c} h\left(PackID\;|\;NodeID_{N}\;|\;rec_{N_{i}}\right)=H. \end{array}$$

If equality (4) is fulfilled in one of these verifications, then the node *n* is a winner. We denote by *Prob*_*h*_ the probability that a hash on *PackID* concatenated with *NodeID* and the receipts *rec*_*Ni*_ that child nodes send to a relaying node collide with a value *H*:

(5) $$\begin{array}{c} Prob_{h}=Prob\left[h\left(PackID\;|\;NodeID_{N}\;|\;rec_{Ni}\right)=H\right]. \end{array}$$

It is assumed that a node can receive only one reward. The probability of a relaying node winning a prize *Prob*_*P*_ in forwarding packets to *N*_*c*_ nodes, where it received the packet from a number of nodes *N*_*f*_, can be defined as follows:

(6) $$\begin{array}{c} Prob_{P}=\left(N_{c}\cdot N_{f}\right)\cdot Prob_{h}. \end{array}$$

As showed in (6), a node can get a reward if it computes the hash of the packet with the receipts from some of its children and gets a winning code. Furthermore, a node can get also a reward if it sends the winner receipt to its father. Therefore, a node can transmit packets or receipts to get an award. Hence, the greater the number of retransmissions is, the greater the probability of winning is. In this way, nodes are motivated to cooperate. Moreover, this mechanism will motivate child nodes to send the receipts to node *N*. However, if we analyse the probability, we find that, when a node gets a winner receipt, it will not broadcast more packets since the previous function is restricted to an only one reward for winner. This behaviour would not be desirable since the objective is to motivate relaying all packets. In order to solve this problem, we propose the use of hash function with nonnegligible probability of collision, which leads to the possibility of having more than one winner receipt for each packet:

(7) ∃i≠j:h(PackID ∣ NodeIDN ∣ recNi)  =h(PackID ∣ NodeIDN ∣ recNj)=H.

In this case, a node could win the lottery for each packet it relays. Thus, nodes can win more than once with the same packet. On the other hand, nodes can also win the lottery with every receipt they return to their parent node. Similar to the previous case, nodes could win the lottery with one or more receipts. Hence, the problem that once a node wins the award it could stop retransmitting the same packet is solved because if a node wins a prize, this does not mean that it cannot win another prize.

#### 4.2.2. The Leader Problem

It is not difficult to assume that no node wants to be leader because in such a case the number of packets it would have to handle is greater than being any other node belonging to the group. However, if we analyse the used mechanisms, we will conclude that in both cases being a leader provides a greater reward than being another node in the group.

According to the introduced group structure, a leader receives all the packets in its group. Hence, it will have a bigger probability to receive receipts that produce a hash collision with *H* so that it could be a winner node. The probability of a leader to win a prize in a group consisting of |*G*| nodes, which receive the packet from one node of the group, could be defined as follows:

(8) $$\begin{array}{c} \left|G\right|\cdot Prob_{h}. \end{array}$$

Therefore, this provides an incentive for leaders to propagate packets because the higher the number of retransmissions, the greater the probability of winning a reward. As a leader, the packets are broadcast to all members of its group, the model promotes that nodes prefer to be leaders, and consequently this mechanism motivates nodes to become leaders and cooperate.

### 4.3. Battery Consumption

In order to ensure proper network functionality, it is necessary to provide users with updated information. Thus, the nodes must cooperate in the delivery of events to all nodes that are met during their life on the network.

If users detect that a mobile phone application consumes too much battery and it does not allow a normal use of the phone, they will turn off the application. This situation would seriously affect the performance of the network. Hence, users must be motivated to keep VAiPho application turned on as much as possible. An advantage of VAiPho regarding battery consumption compared to other existing mobile phone applications is that VAiPho does not use any 3G connections, which is currently the largest battery consuming service. Besides, the offer of an incentive mechanism, the reception of useful information in real time, and the possibility to choose a battery level below which the application automatically stops working are three VAiPho features that motivate users to keep their devices turned on as long as possible.

**Figure 6:**
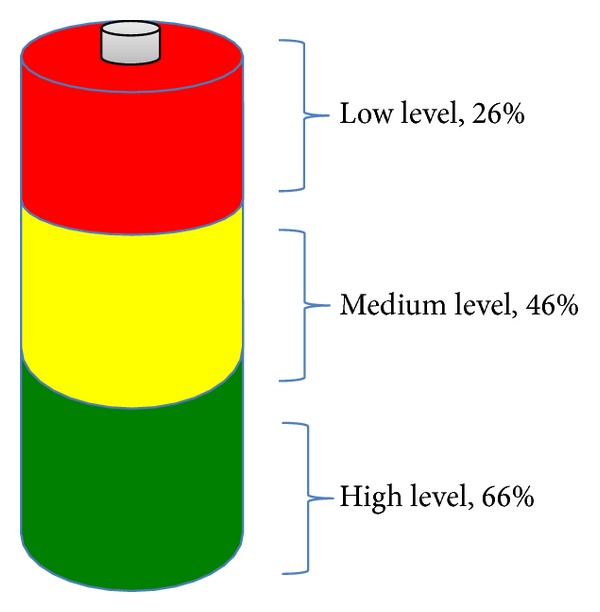
Limit of battery level.

Three battery levels are here proposed for the decision about whether to cooperate in relaying packets or not. The user may establish these levels, which, as shown in Figure 6, in the implementation, have been defined: high (66%), medium (46%), and low (26%). Thus, when the battery level drops below the threshold selected by the user called limit of battery level (LBL), nodes do not cooperate in packet forwarding and other network operations, and the remaining level of battery is used for other purposes.

In order to determine the approximate battery consumption involved in running VAiPho, we made a study with real devices. Considering that the used services that represent the highest battery consumption on the application are GPS, Wi-Fi, and Bluetooth, we analysed and got the following data. GPS has a consumption of about 25% battery every hour, while the use of Wi-Fi and Bluetooth gives a sum of 10% every 8 hours, representing a total of 1.25% per hour. Taking these data as a starting point, we made some simulations. In particular, nodes with different battery levels were considered. In the first case, all nodes initially started the simulation with a high level of battery between 75 and 100%; in the second case, they had medium levels between 46 and 75%, and the third case was with low battery levels: 25–46%. Moreover, nodes had different LBL uniformly distributed according to the three levels defined above. During the simulation, nodes consumed battery, so when they reached their chosen LBL, they turned off the VAiPho application and did not cooperate more. The main aim of the simulation consisted in analyzing the network performance for three different cases. The first one is a simulation where nodes are in an optimal case because they have very high levels of battery. The worst scene is where all nodes start with very low battery level. Finally, an intermediate state is also considered.

**Figure 7:**
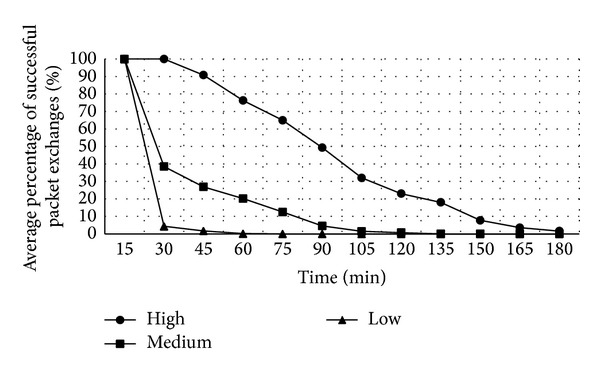
Battery consumption of VAiPho.

Figure 7 shows the relationship between the time of application usage without recharging the battery and the average percentage of successful packet exchange, depending on the three defined battery levels. In the simulations, the number of nodes was 100, and about 1000 connections were established every 15 minutes. In particular, Figure 7 shows the averages obtained from simulations. In particular, in every case, the battery level set by the users in order to stop VAiPho was as follows:

- when the battery drops below the level considered high, for 33 nodes,
- when the battery drops below the level considered medium, for 33 nodes,
- when the battery drops below the level considered low, for 34 nodes.

As we can see in Figure 7, the percentage of successful connections decreases as battery decreases. The worst case is when all nodes have initially very low battery level. However, considering that only 34 vehicles or fewer were running the application, because the remaining 66 stopped it according to their stipulated battery level threshold, the results were pretty good. In particular, the simulations showed that users could freely set their preferred thresholds to stop VAiPho without compromising the network performance, so it may be considered an effective countermeasure to prevent noncooperation. Besides, taking into account that the average trip time by car is about 22 minutes each way, reaching 50% already after 90 minutes is a sufficiently high percentage that guarantees an appropriate quality of the offered services.

Although the results regarding battery consumption shown by the simulations are good, the use of GPS with a 25% of battery consumption per hour could be considered inadequate for the proposal if the application has to run for a long time with no recharge. A solution to this problem can be based on the proposal presented in [22] based on the use of Wi-Fi and cell tower triangulation for location, which provide fast and lower energy location methods.

### 4.4. Storage Space

A decision about whether to store or to drop packets requires taking into account several complex features such as traffic density, type of road (conventional or highway), and time (rush hours). These measures are quite complex and would require a scheme to implement some artificial intelligence method. However, we can get advantages of the group-based proposal where nodes belong to a group and a leader node exists in each group.

Besides, in order to ensure an optimal use of storage space, we define two decision parameters about whether to store or discard a packet: time and distance. These parameters are used to decide whether to save or delete packets from storage space, which prevents the fact that events can remain stored indefinitely.

The time parameter implies that the source node has to add a timestamp to each event. This timestamp indicates the moment when it detected and/or generated a packet. It prevents events from remaining indefinitely stored in memory because they are removed from storage space when they expire. This happens when the time exceeds a threshold. The source node sets this threshold* Texpiration* according to a radius *R* and its speed as defined below:

(9) $$\begin{array}{c} \frac{R}{speed}\cdot 60={Texpiration}_{\min}. \end{array}$$

This radius *R* depends on the type of event *E* and the* speed*. In most cases, information generated at a certain location in a VANET is not interesting out of a radius distance. For example, the event might be a free parking lot space in the centre of a city, which would not be interesting for a driver that is two miles away. However, if the event were about a traffic jam, then the information would be very helpful for the same driver. This means that the basic radio *R* must be greater or lesser depending on the type of event.

**Figure 8:**
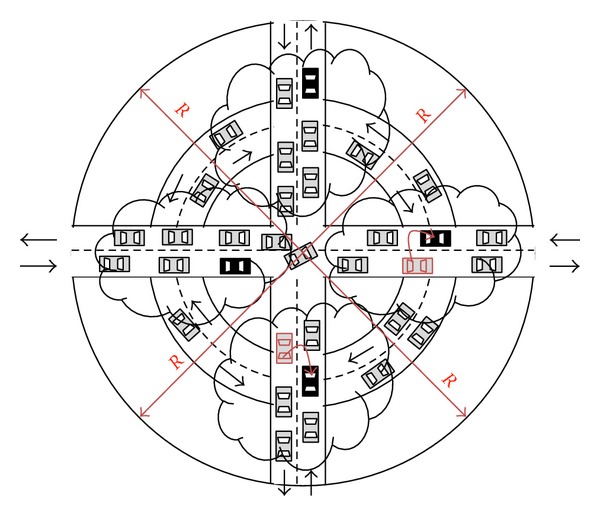
Event generation and action range.

It is possible that nodes remain indefinitely inside the same radius so a time parameter is necessary to delete old packets. Besides the radio, the node speed also influences the total time that must be considered for event storage. This is due to the fact that a vehicle does not use the same period of time travelling on a highway as on a conventional road, as speed is completely different. Therefore, time of storage expiration for each event must depend both on radius and on speed.  Figure 8 shows the scheme idea through an example of the radius-based idea.

Vehicles leaving the radius zone will drop the packet. However, those nodes that stay inside the radio will keep carrying and forwarding the packet to the vehicles they meet. The operating flow of the proposal scheme depends on the role of the nodes.

**Figure 9:**
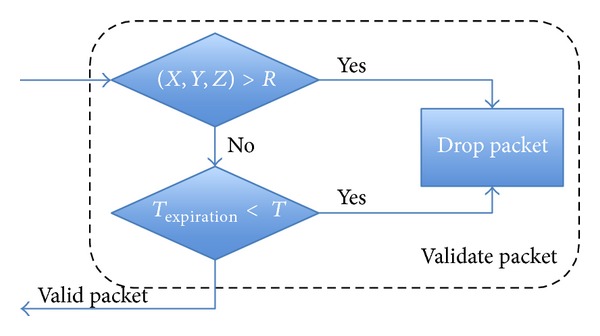
Checking event.

**Figure 10:**
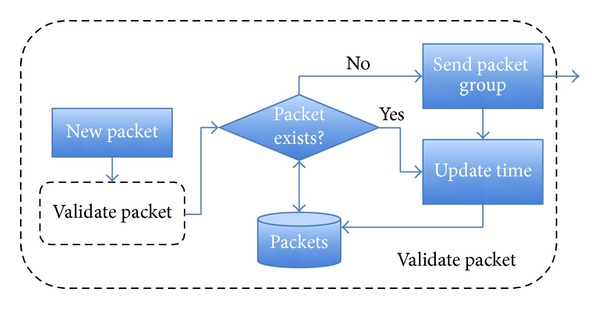
Leader flow when a packet is received.

**Figure 11:**
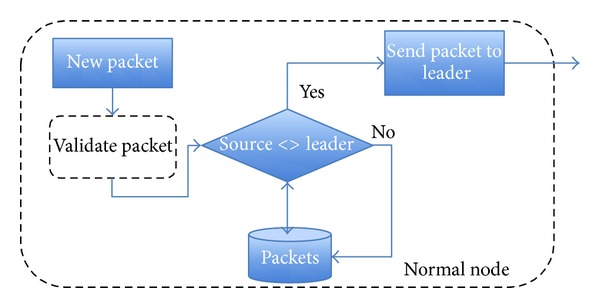
Node flow when a packet is received.

- *Leader Node*. In the proposal, when a leader receives a packet, before making a decision, it validates the information.  Figure 9 illustrates the routing flow algorithm for validation. First, it checks whether its position is inside the radius* R*; then, it checks the expiration time. If both alternatives are correct, the flow carries on the next step. Otherwise, it drops the message. In order to avoid unnecessary message forwarding, the leader has to check whether this information has just been sent to nodes in its group, and, in such a case, the leader does not store and relay the information. On the other hand, if the information is new or the timestamp has been updated, the leader will forward the message and store the new information. Besides, if the leader detects a new vehicle in its group, it sends all the updated information that is stored in the database (see Figure 10).
- *Normal Node*. A normal node has two operation modes. It could receive a packet from the leader or from a normal node. In the first case, it validates the information, checks the source node, and stores the information in its database. In order to minimize the broadcast overhead, in the second option, it validates the information. If the validation is right, it sends the packet to the leader, which will send the new packet to all nodes inside its group.  Figure 11 illustrates this flow. Finally, when a node receives the information from the leader, it stores it in its database.

## 5. Conclusions

This paper has addressed the cooperation issue in fully distributed and decentralized VANETs. Several countermeasures to ensure cooperative behaviour have been proposed. In order to ensure that road traffic information available to drivers is trustful, a new cooperative scheme that allows generating true event warning packets has been described here. It was demonstrated that not only it provides truthful information but it also reduces the number of generated packets and hence the computational complexity. Besides, a new incentive scheme inspired by the lottery idea was proposed to encourage nodes to be relay information. Finally, different aspects that can lead to failure in cooperation, such as storage or battery consumption, have been taken into account when designing the proposal. We have evaluated the performance of cooperative groups when generating events and detecting misbehaviour, and the results confirm that this is a promising approach for increasing channel efficiency and decreasing false information generation in vehicular ad hoc networks.

## References

[1] Caballero-Gil P, Caballero-Gil C, Molina-Gil J (2013). Design and implementation of an application for deploying vehicular networks with smartphones. *International Journal of Distributed Sensor Networks*.

[2] Caballero-Gil P, Caballero-Gil C, Molina-Gil J System for securely communicating in a spontaneous self-managed vehicular ad-hoc network.

[3] Buttyán L, Hubaux J (2003). Stimulating cooperation in self-organizing mobile Ad Hoc networks. *Mobile Networks and Applications*.

[4] Lee S, Pan G, Park J, Gerla M, Lu S Secure incentives for commercial ad dissemination in vehicular networks.

[5] Feng L, Jie W FRAME: an innovative incentive scheme in vehicular networks.

[6] Lo NW, Tsai HC (2009). A reputation system for traffic safety event on vehicular AD Hoc networks. *EURASIP Journal on Wireless Communications and Networking*.

[7] Schmidt RK, Leinmüller T, Schoch E, Held A, Schäfer G Vehicle behavior analysis to enhance security in VANETs.

[8] Dotzer F, Fischer L, Magiera P VARS: a vehicle Ad-Hoc network reputation system.

[9] Wang Z, Chigan C (2007). Cooperation enhancement for message transmission in VANETs. *Wireless Personal Communications*.

[10] Raya M, Papadimitratos P, Aad I, Jungels D, Hubaux JP (2007). Eviction of misbehaving and faulty nodes in vehicular networks. *IEEE Journal on Selected Areas in Communications*.

[11] Sun J, Fang Y A defense technique against misbehavior in VANETs based on threshold authentication.

[12] Frese C, Beyerer J, Zimmer P Cooperation of cars and formation of cooperative groups.

[13] Batz T, Watson K, Beyerer J Recognition of dangerous situations within a cooperative group of vehicles.

[14] Aikebaier A, Enokido T, Takizawa M (2011). Trustworthy group making algorithm in distributed systems. *Human-Centric Computing and Information Sciences*.

[15] Mousannif H, Khalil I, Al Moatassime H (2011). Cooperation as a service in VANETs. *Journal of Universal Computer Science*.

[16] Xiong N, Vasilakos AV, Yang LT, Pedrycz W, Zhang Y, Li Y (2010). A resilient and scalable flocking scheme in autonomous vehicular networks. *Mobile Networks and Applications*.

[17] Tseng FH, Chou LD, Chao HC (2011). A survey of black hole attacks in wireless mobile ad hoc networks. *Human-Centric Computing and Information Sciences*.

[18] Raya M, Aziz A, Hubaux J Efficient secure aggregation in VANETs.

[19] Caballero-Gil P, Caballero-Gil C, Molina-Gil J (2013). How to build vehicular ad-hoc networks on smartphones. *Journal of Systems Architecture*.

[20] Molina-Gil JM, Caballero-Gil P, Hernández-Goya C, Caballero-Gil C Data aggregation for information authentication in VANETs.

[21] Jakobsson M, Hubaux J-P, Buttyan L A micro-payment scheme encouraging collaboration in multi-hop cellular networks.

[22] Lin K, Kansal A, Lymberopoulos D, Zhao F Energy-accuracy trade-off for continuous mobile device location.

